# Supplementary material for: MetaRibo-Seq measures translation in microbiomes
Source: Nat Commun. 2020 Jun 29;11:3268. doi: 10.1038/s41467-020-17081-z (PMC7324362; doi:10.1038/s41467-020-17081-z)
Supplement: Supplementary file 10 — Supplementary Data 7 [file 41467_2020_17081_MOESM10_ESM.zip › File2/Confidence_High_Taxonomy/305457_out.krona.html]

Javascript must be enabled to view this page.

members
magnitude
magnitudeUnassigned
count
unassigned
taxon
rank

305457\_out

29

superkingdom
2
29

29
1239
phylum

class
29
186801

186802
29
order

family
216572
1

genus
459786
1

species

SRS1055022\_contig\_number\_12499
1945593
1

family
27
186803

genus
26
841

species

SRS023526\_contig\_number\_contig-100\_33122.116973SRS064557\_contig\_number\_22861SRS144183\_contig\_number\_27215
2293144
3

301302
1
species

SRS015431\_contig\_number\_17240

species

SRS011084\_contig\_number\_33622SRS014459\_contig\_number\_26682SRS015854\_contig\_number\_contig-100\_3106.3106SRS017247\_contig\_number\_contig-100\_29147.29147SRS018984\_contig\_number\_contig-80\_8649.8649SRS024549\_contig\_number\_1723SRS050752\_contig\_number\_28669SRS052027\_contig\_number\_contig-100\_33998.33998SRS053335\_contig\_number\_contig-100\_4227.4227SRS056273\_contig\_number\_contig-100\_1259.195395SRS063040\_contig\_number\_contig-100\_12806.53308SRS075341\_contig\_number\_contig-100\_4110.162075SRS077454\_contig\_number\_contig-100\_4563.124336SRS097889\_contig\_number\_contig-100\_3065.3065SRS098827\_contig\_number\_contig-100\_849.92162SRS104975\_contig\_number\_contig-100\_6139.141251SRS1055049\_contig\_number\_7780SRS148721\_contig\_number\_contig-100\_20447.171930SRS149181\_contig\_number\_28266SRS149244\_contig\_number\_contig-100\_640.82514SRS893378\_contig\_number\_20239SRS893380\_contig\_number\_contig-100\_505.34446
166486
22

genus
1
33042

1
410072

SRS147919\_contig\_number\_18272
species

31979
1
family

genus
1485
1

1
1262836

SRS142781\_contig\_number\_9053
species
